# Plasma-Metanephrines in Patients with Autoimmune Addison’s Disease with and without Residual Adrenocortical Function

**DOI:** 10.3390/jcm12103602

**Published:** 2023-05-22

**Authors:** Anna-Karin Åkerman, Åse Bjorvatn Sævik, Per Medbøe Thorsby, Paal Methlie, Marcus Quinkler, Anders Palmstrøm Jørgensen, Charlotte Höybye, Aleksandra J. Debowska, Bjørn Gunnar Nedrebø, Anne Lise Dahle, Siri Carlsen, Aneta Tomkowicz, Stina Therese Sollid, Ingrid Nermoen, Kaja Grønning, Per Dahlqvist, Guri Grimnes, Jakob Skov, Trine Finnes, Jeanette Wahlberg, Synnøve Emblem Holte, Katerina Simunkova, Olle Kämpe, Eystein Sverre Husebye, Marianne Øksnes, Sophie Bensing

**Affiliations:** 1 Department of Medicine, Örebro University Hospital, 701 85 Örebro, Sweden; 2 Department of Molecular Medicine and Surgery, Karolinska Institutet, 171 76 Stockholm, Sweden; 3 Department of Clinical Science, University of Bergen, 5021 Bergen, Norway; 4 K.G. Jebsen Center for Autoimmune Disorders, University of Bergen, 7804 Bergen, Norway; 5 Hormone Laboratory, Department of Medical Biochemistry and Biochemical Endocrinology and Metabolism Research Group, Oslo University Hospital, 0372 Oslo, Norway; 6 Institute of Clinical Medicine, University of Oslo, 0372 Oslo, Norway; 7 Department of Medicine, Haukeland University Hospital, 5009 Bergen, Norway; 8 Endocrinology in Charlottenburg, 10627 Berlin, Germany; 9 Department of Endocrinology, Oslo University Hospital, 0372 Oslo, Norway; 10 Department of Endocrinology, Karolinska University Hospital, 171 76 Stockholm, Sweden; 11 Department of Medicine, Vestfold Hospital Trust, 3103 Tønsberg, Norway; 12 Department of Internal Medicine, Haugesund Hospital, 5528 Haugesund, Norway; 13 Department of Endocrinology, Stavanger University Hospital, 4068 Stavanger, Norway; 14 Department of Medicine, Sørlandet Hospital, 4604 Kristiansand, Norway; 15 Department of Medicine, Drammen Hospital, Vestre Viken Health Trust, 3004 Drammen, Norway; 16 Department of Endocrinology, Akershus University Hospital, 1478 Lørenskog, Norway; 17 Department of Public Health and Clinical Medicine, Umeå University, 901 87 Umeå, Sweden; 18 Division of Internal Medicine, University Hospital of North Norway, 9038 Tromsø, Norway; 19 Tromsø Endocrine Research Group, Department of Clinical Medicine, UiT the Arctic University of Norway, 9037 Tromsø, Norway; 20 Section of Endocrinology, Innlandet Hospital Trust, 2381 Hamar, Norway; 21 School of Medical Sciences, Faculty of Medicine and Health, Örebro University, 702 81 Örebro, Sweden; 22 Department of Medicine, Sørlandet Hospital, 4838 Arendal, Norway; 23 Department of Medicine (Solna), Karolinska University Hospital, Karolinska Institutet, 171 76 Stockholm, Sweden

**Keywords:** Addison’s disease, adrenal cortex, adrenal medulla, catecholamines, metanephrines, residual function

## Abstract

Purpose: Residual adrenocortical function, RAF, has recently been demonstrated in one-third of patients with autoimmune Addison’s disease (AAD). Here, we set out to explore any influence of RAF on the levels of plasma metanephrines and any changes following stimulation with cosyntropin. Methods: We included 50 patients with verified RAF and 20 patients without RAF who served as controls upon cosyntropin stimulation testing. The patients had abstained from glucocorticoid and fludrocortisone replacement > 18 and 24 h, respectively, prior to morning blood sampling. The samples were obtained before and 30 and 60 min after cosyntropin stimulation and analyzed for serum cortisol, plasma metanephrine (MN), and normetanephrine (NMN) by liquid-chromatography tandem-mass pectrometry (LC-MS/MS). Results: Among the 70 patients with AAD, MN was detectable in 33%, 25%, and 26% at baseline, 30 min, and 60 min after cosyntropin stimulation, respectively. Patients with RAF were more likely to have detectable MN at baseline *(p* = 0.035) and at the time of 60 min (*p* = 0.048) compared to patients without RAF. There was a positive correlation between detectable MN and the level of cortisol at all time points (*p* = 0.02, *p* = 0.04, *p* < 0.001). No difference was noted for NMN levels, which remained within the normal reference ranges. Conclusion: Even very small amounts of endogenous cortisol production affect MN levels in patients with AAD.

## 1. Introduction

The adrenal glands are vital for the regulation of both the endocrine stress response and body homeostasis, mediated by adrenocortical steroids from the outer cortex and catecholamines from the inner medulla. Although the cortex and the medulla originate from different embryological tissues, their anatomical proximity is not at random, as normal functions in both layers are interdependent [1,2,3,4]. For instance, cortisol from the adrenal cortex is important for adrenomedullary organogenesis and catecholamine synthesis, i.e., epinephrine and norepinephrine [5,6,7]. Regulation of steroidogenesis is in turn dependent on catecholamines [8]. When exposed to stress, concomitant secretion of glucocorticoids (GCs) from the adrenal cortex and epinephrine from the adrenal medulla occur through the bidirectional regulation of production and release. In this process, phenyl ethanolamine-N-methyltransferase (PNMT) is the key enzyme responsible for the synthesis of epinephrine from norepinephrine, and PNMT levels and activity are dependent on the local production of GCs in the adrenal cortex [7,9,10,11]. Metanephrine (MN) and normetanephrine (NMN) are metabolites of catecholamines. While most plasma MN is produced in the adrenal medulla, the same is true for only about 20 percent of plasma NMN. Instead, most plasma NMN originates from norepinephrine released by sympathetic nerves or the extraneuronal metabolism of norepinephrine [12].

Primary adrenocortical insufficiency provides an opportunity to further explore adrenomedullary function in a low or GC-depleted state. In congenital adrenal hyperplasia (CAH), low levels of epinephrine are reported from birth through adulthood [13,14], and patients also show significantly lower epinephrine response during moderate–intense physical activity [15]. Similarly, impaired epinephrine secretion in response to hypoglycemia has been described in patients with secondary adrenal insufficiency [16,17,18]. Zuckerman-Levin et al. studied individuals with isolated GC deficiency due to adrenocorticotropic hormone (ACTH) unresponsiveness, suggesting lower physical performance in these patients due to altered levels of epinephrine and norepinephrine [16,17,18,19]. Patients with autoimmune Addison’s disease (AAD) [20,21,22] are reported to have lower levels of epinephrine during rest as well as after strenuous physical activity and a reduced capacity for exercise compared to healthy individuals [23]. The limitation in physical capacity might partly be caused by the impaired epinephrine production. GC replacement therapy does not seem to normalize catecholamine levels [24].

Until recently, it was assumed that all patients with AAD over time develop total loss of adrenocortical function. In a recent study, we showed that one third of patients with AAD produce low levels of GCs even years after diagnosis [25], as indicated also by others [26,27]. Whether this residual adrenocortical function [28] is of significance for adrenomedullary function is currently unknown.

Here, we aimed to investigate adrenomedullary function in relation to adrenocortical function. We explored if the degree of residual GC-production correlated with levels of plasma metanephrines and whether there was a difference in basal and ACTH-stimulated levels of plasma metanephrines in AAD patients with and without RAF.

## 2. Materials and Methods

### 2.1. Patients

The inclusion and exclusion criteria are described in detail elsewhere [25]. In short, RAF was defined as quantifiable levels of S-cortisol (<0.91 nmol/L) and S-11-deoxycortisol (0.11 nmol/L). Here, we included all 50 patients with verified RAF as well as 20 patients without RAF who served as controls upon undertaking a cosyntropin test in the previous study, yielding a total of 70 patients with AAD. The basic characteristics are shown in Table 1.

### 2.2. Cosyntropin Testing and Metanephrine Assay

In short, all of the participants went through a standard 250 ug cosyntropin test, with samples taken at baseline (0 min) and after 30 and 60 min. Before sampling, the patients abstained from cortisone acetate or hydrocortisone and fludrocortisone for at least 18 and 24 h, respectively. Analysis of plasma MN and plasma NMN was performed by the liquid chromatography-tandem mass spectrometry (LC-MS/MS) method [29,30] at the Hormone Laboratory, Oslo University Hospital, Norway. The normal reference interval for plasma MN was <0.34 nmol/L and the lower level for detection was ≥0.1 nmol/L (CV 12% at 0.29 nmol/L). For plasma NMN, the reference intervals were age specific 16–39 years < 0.63 nmol/L, 40–59 years < 0.76 nmol/L, and >60 years < 1.2 nmol/L, and the lower level for detection was ≥0.2 nmol/L (CV 10% at 0.68 nmol/L). The analytical CV% was a maximum of 20 at the lower level of detection. Both methods are accredited according to NS-EN ISO/IEC 17025.

### 2.3. Statistics

The descriptive statistics are presented as numbers and percentages for categorical data and as the medians and range (interquartile range, IQR) or the mean with the standard deviations as appropriate. We compared the characteristics between the groups with and without RAF. Wilcoxon rank-sum tests or two-sample t-tests were applied to compare continuous variables, and Pearson’s chi-square test was used to compare the categorical variables. The correlations between RAF and the probability of detectable MNs at baseline, 30 min, and 60 min were estimated using logistic modelling with a robust measure. All the statistical analyses were performed in Stata MP 17.1. The alpha value was set to 0.05.

### 2.4. Ethics

Ethical permission was granted by the Regional Ethical Committee of South-East Norway (permit no. 2018/751/REK Sør-Øst), of Stockholm, Sweden (permit no. 2018/2247-32) and the Regional Ethical Committee of Berlin, Germany (permit no. Eth-47/18). Written informed consent was obtained from all participants.

## 3. Results

**Figure 1:**
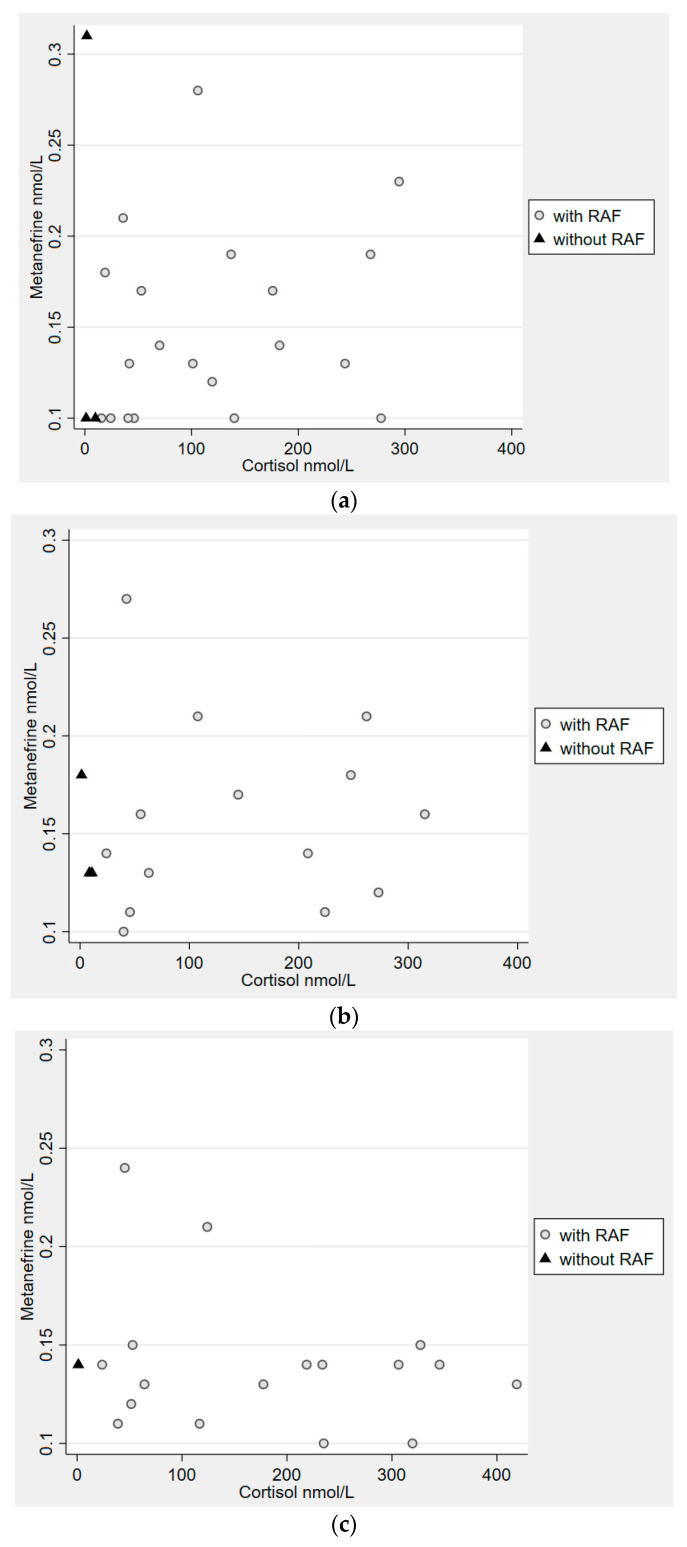
(**a**) Distribution of cortisol and metanephrine at baseline in patients with detectable MN. P-metanephrine and s-cortisol, both in nmol/L; detectable level of MN ≥ 0.1. (**b**) Distribution of cortisol and metanephrine at 30 min, during the cosyntropin test, in patients with detectable MN. P-metanephrine and s-cortisol, both in nmol/L; detectable level of MN ≥ 0.1. (**c**) Distribution of cortisol and metanephrine at 60 min, during the cosyntropin test, in patients with detectable MN. P-metanephrine and s-cortisol, both in nmol/L; detectable level of MN ≥ 0.1.

Plasma MN was detectable, i.e., ≥0.10 nmol/L, in 33% (*n* = 23) at baseline, 25% (*n* = 17) at the time of 30 min, and 26% (*n* = 18) at the time of 60 min (Table 2). In those patients, the distribution of MN and cortisol values at the different time points is depicted in Figure 1a–c. Patients with RAF were more likely to have detectable levels of MN at baseline *(p =* 0.056) and at the time of 60 min (*p* = 0.034) compared to patients without RAF (model not presented).

### 3.1. Comparison of Patients with and without RAF

**Figure 2:**
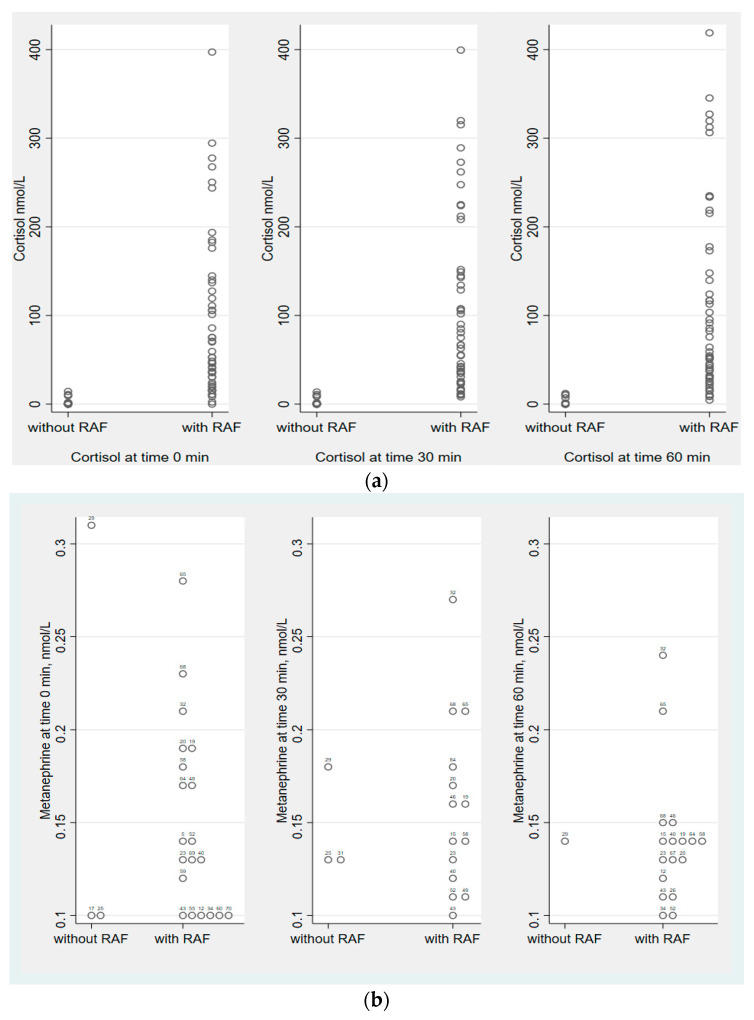
(**a**) Distribution of cortisol levels at 0, 30, and 60 min, during the cosyntropin test, in patients with and without residual adrenocortical function. (**b**) Distribution of detectable metanephrine levels at 0, 30, and 60 min, during the cosyntropin test, in patients with and without residual adrenocortical function.

The patients with RAF had significantly higher systolic blood pressure (BP) (*p* = 0.001) and higher levels of DHEAS (*p* = < 0.001); no other differences in baseline characteristics were seen (Table 1). The distribution of cortisol and MN in patients with and without RAF at 0, 30, and 60 min is shown in Figure 2a,b. MN was detectable in 23 of the 50 patients with RAF. A total of 9 patients had detectable MN at one time point, 3 patients had detectable MN at two time points, and 12 patients had detectable MN at all three time points. In the 20 patients without RAF, MN was detectable in 4 separate patients. Of these, one patient had detectable MN at all three time-points, with the highest MN level (0.31 nmol/L) noted at baseline. There was no increase in MN during the cosyntropin test in patients with RAF or in patients without RAF.

### 3.2. Metanephrine and Cortisol

In patients with detectable MN, there was a positive correlation with the level of cortisol at all three time points: baseline (*p* = 0.02), the time of 30 min (*p* = 0.04), and at the time of 60 min (*p* < 0.001). At baseline, we did not find any difference in age (*p =* 0.46), sex (*p* = 0.15), BMI (*p* = 0.83), frequency of adrenal crisis (*p =* 0.81), systolic BP (*p* = 0.50), diastolic BP (*p* = 0.43), or disease duration (*p =* 0.97) in the patients with detectable MN compared to those without. However, significantly more men than women had detectable MN at the times of 30 and 60 min (*p* = 0.008, *p* = 0.02).

### 3.3. Normetanephrine

NMN was detectable in most patients and no significant differences in NMN levels were found either between the subgroups at baseline nor after the cosyntropin test. The NMN levels (0.52, 0.56, and 0.58 nmol/L, median, IQR) were within the normal ranges in each age group (16–39, 40–59, and > 60 years), respectively.

## 4. Discussion

This is the first study to report MN and NMN levels in patients with AAD in relation to RAF [25]. Patients with RAF were more likely to have detectable MN before as well as after the cosyntropin test. Even though the residual production of cortisol in absolute values was generally very low, a correlation between MN and cortisol was demonstrable.

ACTH is thought to regulate epinephrine synthesis indirectly by inducing GC secretion from the adrenal cortex. The endogenous GCs in turn stimulate the PNMT activity needed for norepinephrine conversion to epinephrine, which is then metabolized to MN [31]. We have previously reported a small increase in GC levels upon completion of the cosyntropin test in patients with RAF [25]. In the current study, we did not find any significant change in MN or NMN levels after the cosyntropin test in either group, suggesting that isolated ACTH stimulation is of little importance to increasing adrenomedullary epinephrine production. Thus, it is possible that any direct or indirect stimulatory role of ACTH on MN and NMN levels had already been fully exploited before the cosyntropin test.

Our findings point to the importance of endogenous cortisol/GC for catecholamine synthesis in the adrenal medulla [5,7], as previously indicated by studies on adrenocortical and adrenomedullary function in patients with CAH. Patients with CAH and severe salt-wasting disease have lower levels of both cortisol, epinephrine, and MN compared to patients with simple virilizing disease as well as healthy controls [32,33]. This is consistent with our patients who suffer from AAD and have impaired production of MN, which seems to be related to endogenous GC deficiency. A previous small study on patients with CAH and healthy controls found a significant increase in both norepinephrine and NMN after a standardized bicycle exercise, suggesting a preserved ability in CAH to mobilize the adrenal medulla upon stress [34]. This contrasts to our findings, but then the stressors are not the same in these studies.

The endogenous GC levels in RAF are generally low [25], and the modest increase in cortisol upon completion of the cosyntropin test might be too small to facilitate any increase in MN levels. In all the patients, the NMN levels were within the normal ranges, but compared to the results in previous studies establishing reference levels for plasma NM, the median in our study tends to be higher [29]. Increased levels of NMN are also found in adrenalectomized patients. This effect is thought to be compensatory because of lower epinephrine levels, leading to changes in norepinephrine production in the sympathetic nerves [35]. This resembles our patients who have a similar pattern in their catecholamine production. Analysis of the clinical consequences of impaired MN production was not included in this study, but it might be part of the explanation for the often-present poor capacity to manage stress.

In this study, patients with RAF had higher baseline systolic BP compared to patients without RAF. In our previous study including a larger number of patients without RAF, we did not detect this difference [25], possibly indicating selection bias in the current study.

To conclude, in patients with AAD, detectable MN is positively correlated to the level of serum cortisol and is more pronounced in patients with RAF, suggesting that even partly preserved endogenous production of cortisol is of importance for MN production. Any clinical implications of this remain to be determined.

## Figures and Tables

**Table 1:** Patient characteristics.

Characteristic		without RAF	with RAF	*p*-Value
N		20	50	
Age (year), median (IQR)		49 (32, 55)	50 (36, 59)	0.62
Female		13 (65%)	23 (46%)	0.15
BMI (kg/m^2^), mean (SD)		24 (4.2)	26 (4.3)	0.20
Adrenal crisis ever	no	5 (25%)	18 (36%)	0.38
	yes	15 (75%)	32 (64%)	
Systolic BP (mmHg), median (IQR)		110 (101,120)	121 (116,132)	0.01
Diastolic BP (mmHg), median (IQR)		71 (69, 80)	78 (70, 85)	0.14
PRC. (mIE/L) median (IQR)		71 (17, 192)	91 (27, 206)	0.25
S-DHEAS (nmol/L) median (IQR)		<0.62 * (0, 192)	428 (160, 628)	<0.001

Abbreviations: BMI; body mass index, BP; blood pressure, IQR; inter-quartile range, S-DHEAS; serum dehydroepiandrosterone sulfate, PRC; plasma renin concentration * Lower limit of quantification.

**Table 2:** Median and range of cortisol * in patients with and without detectable metanephrine ** levels at 0, 30, and 60 min in patients with and without residual adrenocortical function during the cosyntropin test.

	All Patients	Cortisol		Without RAF ***	Cortisol		With RAF	Cortisol	
	N	Median	Min-Max	N	Median	Min-Max	N	Median	Min-Max
Time 0 min									
MN < 0.1	47	16	<0.91–397	17	<0.91	<0.91–14	30	47	<0.91–397
MN ≥ 0.1	23	70	1–294	3	1.8	1–10	20	104	16–294
Time 30 min									
MN < 0.1	52	25	<0.91–399	17	<0.91	<0.91–14	35	67	8–399
MN ≥ 0.1	17	63	1–315	3	8.5	1–11	14	126	24–315
Time 60 min									
MN < 0.1	51	18	<0.91–312	19	<0.91	<0.91–2	32	47	5–312
MN ≥ 0.1	18	151	1–418	1	1	1	17	178	24–418

* P-metanephrine and s-cortisol, both in nmol/L ** Detectable level of MN ≥ 0.1, *** RAF = residual adrenocortical function.

## Data Availability

The datasets generated and/or analyzed during the current study are not publicly available but are available from the corresponding author upon reasonable request.

## Trial Registration

CinicalTrials.gov registration number: NCT03793114 (6 November 2018).

## Abbreviations

AAD	Autoimmune Addison’s disease
ACTH	Adrenocorticotropic hormone
AD	Aldehyde dehydrogenase
BP	Blood pressure
CAH	Congenital adrenal hyperplasia
COMT	Catechol O-methyltransferase
DBH	DOPA β-hydroxylase
DDC	DOPA decarboxylase
GC	Glucocorticoid
LC-MS/MS	Liquid chromatography–tandem-mass spectrometry
MAO	Monoaminoxidase
MN	Metanephrine
NMN	Normetanephrine
PAH	Phenylalanine hydroxylase
PNMT	Phenylethanolamine-N-methyltransferase
RAF	Residual adrenocorticoid function
TH	Tyrosine hydroxylase
